# Long-term Functional Outcomes After Operatively Treated Unimalleolar, Bimalleolar, and Trimalleolar Ankle Fractures: A 15-22-Year Follow-up Study of 125 Patients

**DOI:** 10.1177/10711007251361509

**Published:** 2025-09-08

**Authors:** Anne Scheuer, Fabian T. Spindler, Judith Schrempf, Wolfang Böcker, Hans Polzer, Sebastian F. Baumbach

**Affiliations:** 1 Department of Orthopaedics and Trauma Surgery, Musculoskeletal University Center Munich (MUM), University Hospital, LMU Munich, Germany; 2 OrthoPlus, Munich, Germany

**Keywords:** ankle fracture, surgery, patient-reported outcome, long-term follow-up, quality of reduction

## Abstract

**Background:**

Despite considerable improvements in surgical treatment strategies for unstable ankle fractures, long-term follow-up studies on conventional treatment strategies are missing. The aim of the study was to assess the patient-reported long-term outcome (≥15 years) following surgically treated ankle fractures.

**Methods:**

Retrospective, single-center, outcome study with a current follow-up. Identified were all patients treated surgically for an unstable ankle fracture between January 2003 and October 2009. Treatment was performed according to the AO principles. General demographics, injury-, fracture- and treatment details, as well as the current patient-reported outcome (Olerud-Molander Ankle Score [OMAS], the Foot and Ankle Ability Measure [FAAM], the European Foot & Ankle Society Score [EFAS], and the EuroQoL–5 dimensions, 5 levels [EQ-5D-5L]) were assessed.

**Results:**

Of 398 eligible patients, 125 patients (31.4%; 54% female, 48% left side) were included. The average age at trauma was 43.1 ± 11.8 years, and the mean follow-up 17.8 ± 2.0 years. Overall, 43%, 22%, and 34% sustained a unimalleolar, bimalleolar, and trimalleolar fracture, respectively. In addition, 53% had a fracture to the posterior malleolus. The overall median outcome for the OMAS, FAAM daily, and EFAS daily were 100 (interquartile range [IQR] 15), 99 (IQR 7), and 96 (IQR 16), respectively. The EQ-5D-5L scored at a median of 1.00 (IQR 0.09). The number of malleoli fractured was the only factor affecting all outcome scores. A moderate or poor outcome was reported for 5% of unimalleolar, 18% of bimalleolar, and 30% of trimalleolar fractures.

**Conclusion:**

Although the overall functional outcome was good, about 15% of patients had considerable impairment. The only factor associated with the patient-reported outcomes was the number of malleoli fractured, with 30% of patients with a trimalleolar ankle fracture presenting inferior outcomes.

**Level of Evidence:** Level IV, retrospective case series

## Introduction

Ankle fractures account for approximately 9% of all fractures.^1,2^ Within the last 2 decades, the surgical treatment of ankle fractures has evolved considerably.^3^ These innovations aim at improving the clinical outcome and reducing the risk for post-traumatic osteoarthritis. One would assume that these innovations were driven by long-term follow-up studies that did show a correlation between surgical treatment strategies and impaired long-term outcome.

But most studies that claim to report on a “long-term” follow-up present follow-up periods between 3 years^4^ and 5 years.^5,6^ These follow-up periods are most likely insufficient to detect an impaired outcome because of posttraumatic osteoarthritis. The few long-term follow-up studies of at least 10 years available are limited by the number of patients,^7^ or miss sufficient detail.^8^ Consequently, valid long-term follow-up data of surgically treated ankle fractures is limited.

Therefore, the aim of the current study was to assess the clinical long-term outcome of at least 15 years in patients following surgical treatment for ankle fractures at a single academic level 1 trauma center.

## Materials and Methods

This is a retrospective outcome study that was approved by the local ethics committee of the University Hospital of Munich (No. 20-0955).

### Patient Selection

At the authors’ academic level 1 trauma center, the electronic patient record had been fully implemented by the end of 2002. The authors identified all patients treated surgically for a closed, isolated ankle fracture between January 2003 and October 2009. Patients were identified within the hospital database using one of the following *International Statistical Classification of Diseases and Related Health Problems, Tenth Revision* (*ICD-10*) codes (S82.5, S82.6, S82.7, S82.81, S82.82, S82.88, M84.07, M90.77) and any surgical code (OPS code). All patients ≥18 years of age at the time of trauma and <80 years at the time of follow-up were eligible. The upper age limit was chosen because patients 80 years or older would probably not be able to answer the questionnaires. Inclusion was independent of medical comorbidities. Pre- and postoperative radiographs must have been available. Each eligible patient was contacted and invited to participate in the current study. Patients had to give written consent and complete the patient-reported outcome measures to be included.

### Data Assessed

The data assessed comprised general demographics, injury-, fracture- and treatment details, as well as the current patient-reported outcome measures. The general demographics assessed were age at trauma and follow-up as well as sex. Injury- or fracture-specific data recorded were number of malleoli fractured (uni-, bi-, or trimalleolar) and the AO classification.^9^ The size of the posterior malleolar (PM) fragment was rated on lateral radiographs as percentage of the total depth of the distal tibia.^10^ Treatment details assessed were the use of an external fixature, choice of implants per the malleoli fractured, and syndesmotic fixation. Because of the long-term follow-up, the assessment of complications was only partially possible. As the outpatient documentation was not available, only complications necessitating in-house treatment could be assessed. During the period analyzed (January 2003 and October 2009), any intravenous medication or surgical revision was performed in-house. These data were recorded. Furthermore, all documented comorbidities were assessed. These included comorbidities stated by the patient or any up-to-date documentation available in the system. The comorbidities were rated per the Charlson Comorbidity Index (CCI)^11^ and the modified Functional Comorbidity Index (mFCI).^12^ The CCI is a widely used comorbidity index and includes 19 weighted conditions. The index was predominantly developed to predict mortality.^11-13^ The mFCI is the weighted evolution of the Functional Comorbidity Index.^14^ It is composed of 18 weighted questions and has been developed to better predict the comorbidity-related functional outcome.^12,15^ As the BMI was not available for the vast majority of patients, this question was excluded, and the maximum score was set to 34. The score was converted to percentage to allow for better comparability. Based on the postoperative radiographs, the quality of reduction (QoR) was rated qualitatively from 1 to 6 (1 = best, 6 = worst) by 2 authors independently (J.S., S.F.B.). No quantitative rating, such as step-off measurements, were performed, as a sufficient quantitative analysis of the QoR can only be conducted on computed tomographic (CT) images.^16^

#### Patient-reported outcome measures

Each patient was asked to complete 3 patient-reported outcome measures (PROMs) and 1 quality of life score.

The PROMs assessed were the Olerud-Molander Ankle Score (OMAS),^17^ the Foot and Ankle Ability Measure (FAAM),^18^ and the European Foot & Ankle Society Score (EFAS).^19^ The OMAS^17^ has been designed for the assessment of ankle fracture patients. It comprises 9 parameters and scores from 0 to 100, with higher scores indicating better function. The minimal clinically important difference (MCID >12 months) is 10.5 points. The MCID was calculated based on the data of the RODEO trial, a multicenter randomized controlled trial on syndesmotic screw removal after ankle surgery.^20^ The FAAM^18^ and the EFAS^19^ are more general foot and ankle outcome scores. The FAAM^18^ comprises of 29 items, and scores in 2 domains: activities of daily living (ADL) and sports. The cumulative scoring is usually translated into a percentage ranging from 0 to 100, with higher scores indicating better functional outcomes. Its MCID is 8 points for the ADL and 9 points for the sports domain among general orthopaedic foot and ankle patients undergoing physical therapy over a 4-week period.^18^ The EFAS^19^ comprises 6 questions (daily) and is extended by 4 questions (sports) aiming at sports activities. Each domain scores from 0 to 4, with higher scores indicating better outcome. For higher responsiveness and comparability to the other scores obtained, the values were translated to a percentage score ranging from 0 to 100. In a recent publication^21^ comparing the EFAS to the SEFAS, the authors also assessed the MCID (minimal detectable change at 90% convidence) for the EFAS. The source data were 126 patients undergoing elective surgical treatment of the forefoot, midfoot, hindfoot, or ankle. The authors subdivided the daily domains into pain and physical function. The MCID for pain and physical function was 1.5 and 1.83, respectively. For the current study, the MCID for the daily domain was set to 1.83, that is, 7.6 points when translated to percentage. Because of the high age of the patient cohort, the sport parts (FAAM, EFAS) were not included in the analysis. The individual scores of the PROMs (OMAS/FAAM/EFAS) were grouped into excellent (90-100), good (80-89), moderate (60-79), and poor (<60).

The patients’ quality of life was assessed via the EuroQoL–5 Dimensions, 5 Level version (EQ-5D-5L).^22^ The EQ-5D-5L assesses 5 health domains, that is, mobility, self-care, usual activities, pain and discomfort, and anxiety and depression. The final score is cumulatively calculated and compared to a visual analog scale for subjective appraisal. The MCID in patients with foot and ankle disorders has not been reported, but for hip and knee osteoarthritis patients with 0.32.^23^

### Statistics

Statistical analysis was performed using the jamovi project software, version 2.3.28. The Shapiro-Wilk test revealed no normal distribution for all parameters (PM fragment size [<0.001], OMAS [<0.001], FAAM daily [<0.001], EFAS daily [<0.001], and EQ-5D-5L [<0.001]) but age and follow-up. Therefore, nonparametric testing, χ^2^ tests, Mann-Whitney *U* test, a nonparametric ANOVA with a Tukey post hoc analysis, and a Spearman correlation were used but for the comparison between included and excluded patients for age at trauma or final follow-up. Nonparametric values are given as median and IQR, normally distributed values as mean ± SD. Because of multiple testing, a Bonferroni alpha level correction was performed, setting the level of significance to *P* < .0125. The individual influence of the assessed factors (age at trauma, sex, number of malleoli fractured, posterior malleolar [PM] fracture [binary and size], syndesmotic fixation [binary], QoR, time to follow-up, CCI, mFCI, complication [binary], and revision [binary]) on the outcome parameters was assessed by a multiple regression analysis using generalized linear models to account for non-normal distribution.

## Results

**Figure 1.**
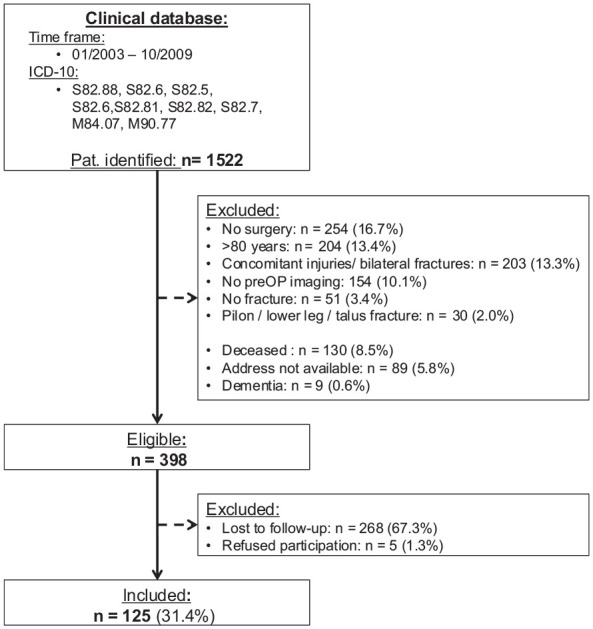
Patient selection flowchart. *ICD-10*, *International Statistical Classification of Diseases and Related Health Problems, Tenth Revision*; Pat., patients; preOP, preoperative.

The initial search resulted in 1522 cases within the author’s clinical database. A total of 398 patients were eligible for further analysis and 125 patients (31.4%) could be included in the final data synthesis (Figure 1).

The cohort characteristics and a comparison to the excluded patients is presented in Table 1. Per the adjusted alpha level, no significant differences were found between the 2 cohorts but the quality of reduction, which was better in the excluded patients (1.7 ± 0.8 vs 2.2.0.9; *P* < .001). For the patients included, the mean time to follow-up was 17.8 ± 2.0 years (range 15.1-22.0 years) and the mean age at trauma 43.1 ± 11.8 years; 54% of patients were female and the left side was affected in 48%. The diagnosis and classification was based on plain radiographs in 111 patients (89%), on CT imaging in 11 patients (9%), and on magnetic resonance imaging in 2 patients (2%). The AO/OTA classification was as follows: A2, 2 patients (2%); B1, 29 patients (23%); B2, 17 patients (14%); B3, 50 patients (40%); C1.1, 2 patients (2%); C1.2, 2 patients (2%); C1.3, 1 patient (1%); C2.1, 5 patients (4%); C2.3, 7 patients (6%); C3.1, 6 patients (5%); C3.3, 1 patient (1%); not classifiable, 3 patients (2%).

**Table 1.** Comparison Between Included and Excluded Patients.

Cofounding Variables	Included (n = 125)	Excluded (n = 273)	*P* Value
Age at trauma, y, mean ± SD	43.1 ± 11.8	40.5 ± 12.3	.056^a^
Age at final follow-up, mean ± SD	61.2 ± 11.7	58.0 ± 12.0	.019^a^
Sex, % female	54	42	.025
Number of malleoli fractured (unimalleolar/bimalleolar/trimalleolar)	43/22/34	48/22/29	.510
PMF, % yes	47	57	.088
PMF size, median (IQR)	18.0 (IQR 24)	18.7 (IQR 19)	.448
Syndesmotic fixation, % yes	37	29	.113
QoR, mean ± SD	2.2 ± 0.9	1.7 ± 0.8	<.001

Abbreviations: IQR, interquartile range; PMF, posterior malleolar fracture; QoR, quality of reduction.

a Independent *t* test.

An external fixature was applied in 7 patients (6%). The lateral malleolus was fractured in 123 (98%) and fixed in 111 patients by one-third tubular plates (97%), intramedullary fixation device (2%), or screws (1%). The medial malleolus was fractured in 54 patients (43%), which was fixed by screw(s) (98%) or tension band wiring (2%). The posterior malleolus (PM) was fractured in 66 patients (53%), 20 of which (30%) were addressed by closed reduction and internal fixation (CRIF) with 1 or 2 anterior-posterior screws. In the remaining 46 patients (70%), the PM fracture was left untreated. The median size of the PM was 13% (IQR 13%) in the untreated and 35% (IQR 15%) in the CRIF group. In 47 patients (38%), an additional syndesmotic stabilization was performed by 1 (72%) or 2 (23%) syndesmotic screw(s) or direct suture (2 patients; 4%). The osteosynthesis was rated as excellent (grade 1) in 23%, good (grade 2) in 42%, sufficient (grade 3) in 26%, and adequate (grade 4) in 8%. The median CCI was 0.43 ± 1.0 (range 0-6), the mFCI was 2.0% ± 4.2% (range 0%-23.5%). Eight patients (6.4%) had a complication, 2 minor complications (asymptomatic non-union and SSI with intravenous antibiotics) and 6 major complications (secondary dislocation [2×], SSI with revision [1×], arthrofibrosis with arthroscopic arthrolysis [3×]). One patient underwent total ankle replacement.

### Patient-Reported Outcome Measures

The overall median outcome for the OMAS was 100 (IQR 15), for the FAAM daily 99 (IQR 7), for EFAS daily 96 (IQR 16), and for the EQ5D-5L 1.00 (IQR 0.09). Still, 17%, 11%, and 19% of the patients had a moderate to poor outcome per the OMAS, FAAM daily, and EFAS daily scores, respectively (Table 2).

**Table 2.** Grouped Outcome per the Obtained PROMs.

	OMAS, %		FAAM daily, %		EFAS daily, %	
Excellent	74	83	81	89	66	80
Good	9		8		14	
Moderate	12	17	7	11	9	19
Poor	5		4		10	

Abbreviations: EFAS, European Foot & Ankle Society Score; FAAM, Foot and Ankle Ability Measure; OMAS, Olerud-Molander Ankle Score; PROMs, Patient Reported Outcome Measures.

**Figure 2.**
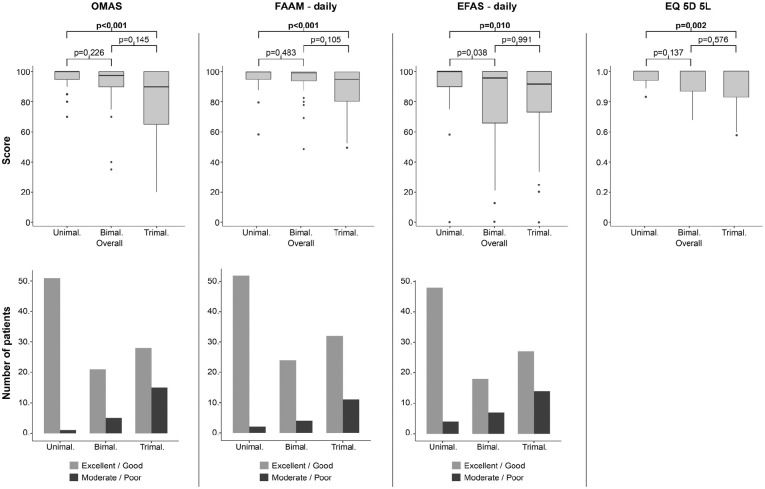
Box plots of the significantly different PROMS per the number of fractured malleoli. Boldface: significant difference. Bimal., bimalleolar; EFAS, European Foot & Ankle Society Score; OMAS, Olerud-Molander Ankle Score; FAAM, Foot and Ankle Ability Measure; Trimal., trimalleolar; Unimal., unimalleolar.

The number of malleoli fractured was the only parameter consistently and significantly affecting the patient-reported outcome (Table 3). Figure 2 (Supplementary Material) provides an overview on the individual results including the post hoc analysis for the number of malleoli fractured. Trimalleolar fractures scored significantly worse than unimalleolar fractures (*P* = .01 to <.001) for all scores assessed. This difference exceeded the MCID for all patient-reported outcomes, that is, the OMAS, FAAM daily, and EFAS daily (Supplementary Material) when considering the mean values. For the median values, only the EFAS daily reached the MCID. A generalized linear regression model revealed the following prediction values: OMAS *R*^2^ = 0.416, FAAM daily *R*^2^ = 0.364, EFAS daily *R*^2^ = 0.378, and EQ-5D-5L *R*^2^ = 0.396.

**Table 3.** Confounding Variables Influencing the PROMs.^a^

Confounding Variables	OMAS	FAAM Daily	EFAS Daily	EQ5D-5L
Age at trauma				
Spearman ρ	−0.214	−0.239	−0.183	−0.233
*P* value	.018	**.007**	.041	**.009**
Age at final follow-up				
Spearman ρ	−0.215	−0.216	−0.181	−0.213
*P* value	.018	.016	.044	.017
Sex, *P* value	.068	.153	.078	.267
Number of malleoli fractured, *P* value	**<.001**	**.001**	**.005**	**.003**
PMF (yes/no), *P* value	.015	.058	.016	.087
PMF size				
Spearman ρ	−0.086	−0.069	−0.058	0.018
*P* value	.500	.579	.641	.887
Syndesmotic fixation (yes/no), *P* value	0.127	0.102	0.091	0.101
QoR, *P* value	.242	.540	.706	.556
CCI				
Spearman ρ	−0.022	−0.016	−0.020	−0.048
*P* value	.821	.856	.831	.598
mFCI				
Spearman ρ	−0.044	−0.012	0.002	−0.049
*P* value	.629	.893	.980	.586
Time of follow-up				
Spearman ρ	0.003	0.102	0.026	0.026
*P* value	.973	.260	.769	.773

Abbreviations: CCI, Charlson Comorbidity Index; EFAS, European Foot & Ankle Society Score; EQ5D-5L, EuroQoL–5 dimensions, 5 level version; FAAM, Foot and Ankle Ability Measure; mFCI, Modified Functional Comorbidity Index; OMAS, Olerud-Molander Ankle Score; PMF, posterior malleolar fracture; PROMs, Patient Reported Outcome Measures; QoR, quality of reduction.

a Boldface: significant difference. Spearman ρ: Spearman rank correlation coefficient.

Finally, a cross-check on possible factors associated to the number of malleoli fractured was performed. Age at trauma (*P* = .067), age at final follow-up (*P* = .076), sex (*P* = .123), syndesmotic fixation (*P* = .943), time to follow-up (*P* = .918), mFCI (*P* = .123), and complications (*P* = .036) did not have a significant influence on the number of malleoli fractured. The only parameters associated to the number of malleoli fractured were the quality of reduction and CCI (Table 4). The PM fracture values were not calculated, as they are inherently biased by the number of malleoli fractured.

**Table 4.** Influence of the Number of Fractured Malleoli on the Postoperative Quality of Reduction and the Carlson Comorbidity Index.^a^

	Unimalleolar Fracture (n = 54)	Bimalleolar Fracture (n = 28)	Trimalleolar Fracture (n = 43)
Quality of reduction	*P* < **.001**		
Grade 1 (n = 29)	44	11	5
Grade 2 (n = 53)	43	46	40
Grade 3 (n = 33)	9	36	42
Grade 4 (n = 10)	4	7	14
Carlson Comorbidity Index	*P* = .**002**		
	0.24 ± 0.91	0.29 ± 0.71	0.77 ± 1.21

a Boldface: significant difference.

## Discussion

The current study assessed the patient-reported outcome after 17.8 ± 2.0 years of 125 operatively treated ankle fractures. Across OMAS, FAAM daily, and EFAS daily, 17%, 11%, and 19% of patients, respectively, demonstrated moderate to poor results, despite high median scores. The only factor significantly associated to the patient-reported outcomes were the number of malleoli fractured, with trimalleolar ankle fractures scoring worst.

Overall, the median outcome across all PROMs assessed ranged between 96 and 100 points. As indicated above, the body of literature on long-term follow-up studies is limited. The authors are aware of 3 studies,^7,8,24^ which are in parts comparable, that is, also including uni-, bi-, and trimalleolar ankle fractures. Lehtola et al^7^ reported on 24 patients with a follow-up of 9.4 (8.9-11) years. Their mean OMAS score was 87-89 points. Kahraman et al^8^ report on 48 patients with a follow-up of 10.8 (10.3-12.5) years. Their mean AOFAS score ranged between 93 and 97 points. Verhage et al^24^ reported on 243 patients with a follow-up of 9.7 (5-17) years. Their median AOFAS was 95 points. All authors rated their overall patient-reported outcomes as good to excellent. These values are in range with the herein reported PROMs.

However, an overall consideration of the outcome scores is misleading. Grouping the individual scores per excellent, good, moderate, and poor revealed that almost 20% of patients had inferior (moderate or poor) patient-reported outcomes in the present study. Moreover, these figures only account for the daily scores in the FAAM and EFAS, which do not rate more strenuous activities. None of the aforementioned studies did a similar analysis. Consequently, although the overall results appear promising, almost 20% of patients do suffer from considerable impairment in their daily lives.

The authors tried to identify factors associated to an inferior outcome. The only factor associated to an impaired patient-reported outcomes (OMAS, FAAM daily, EFAS daily) and on the patients’ quality of life (EQ-5D-5L) were the number of malleoli fractured. The number of malleoli fractured could be considered a surrogate parameter for the injury severity and the force transmitting through the joint. Trimalleolar ankle fractures did not only score significantly worse than unimalleolar fractures, but they also showed a considerable wider IQR. Therefore, the outcome of unimalleolar ankle fractures is not only better but also more reproducible than that of trimalleolar ankle fractures. Verhage et al^24^ showed comparable results, reporting that bi- and trimalleolar fractures led to significantly worse patient-reported outcomes compared with unimalleolar fractures after 10 years of follow-up. In order to account for possible confounders, a generalized linear model was performed. Still the model explained less than 50% of the variance. One of the reasons could be the ceiling effect of the PROMs assessed. A ceiling effect describes the effect that a score cannot sufficiently discriminate good from excellent outcomes and is the reason for the non-normal distribution. Other reasons could be that we are either assessing data insufficiently (injury severity, quality of reduction) or missing out on important confounders.

To make the results comprehensible for our patients, the authors also assessed the proportion of patients with excellent to good and moderate to poor results separately for the number of malleoli fractured. Whereas 98%, 96%, and 92% of unimalleolar ankle fractures had excellent to good results per the OMAS, FAAM daily, and EFAS daily, these numbers cut to 65%, 74%, and 66% for trimalleolar ankle fractures. Consequently, 30% of patients who had suffered a trimalleolar ankle fracture could expect a considerably impaired long-time patient-reported outcome. Future studies should therefore focus on more complex ankle fractures aiming at identifying risk factors for an impaired outcome. Possible factors could be accompanying intra-articular pathologies such as (osteo-)chondral lesion or loose bodies, untreated ligamentous injuries to the syndesmotic or deltoid ligament complex, or an impaired QoR due to the higher complexity of trimalleolar ankle fractures.

Because of the retrospective nature of this study, a subsequent analysis on these factors was limited. Still, the current study revealed that the QoR was significantly worse in trimalleolar ankle fractures, compared with unimalleolar ankle fractures. Whereas the QoR was rated as excellent to good (grade 1 or 2) in 87% of the unimalleolar fractures, this number decreased to only 45% in trimalleolar ankle fractures. An exact analysis of the QoR was not possible on the plain radiographs available but can only be conducted on intra- or postoperative CT imaging. Moreover, patients who had suffered a trimalleolar ankle fracture had a significantly higher CCI compared with unimalleolar ankle fractures.

In the introduction, the authors named possible advances in the surgical strategies of ankle fractures. In the context of the QoR, it will be interesting to see if open reduction and internal fixation (ORIF) of the PM fracture will have an impact on the patient-reported outcomes.^25,26^ In the current study, the “traditional” PM fracture reduction and fixation technique was applied. Thereby, a maximum dorsal flexion of the ankle joint was believed to reduce the PM fragment due to ligamentotaxis. The PM fracture was then fixed by percutaneous (CRIF) anterior-to-posterior screws. More recent evidence has already shown significantly better clinical and radiologic outcomes for ORIF compared with CRIF of the PM fragment.^27^ Whether these results will also impact the long-term outcome has yet to be shown.

Several limitations must be discussed. First, it is a retrospective study with all subsequent limitations, including a loss to follow-up of 69%, a possible selection bias, and missing latest follow-up radiographs to assess possible osteoarthritic changes. Still, other long-term follow-up studies (>10 years) reported loss to follow-up rates of 84%^28^ and 71%.^8^ The high rate of loss to follow-up in combination with a better quality of reduction grade in the lost-to-follow-up group could indicate an overestimation of the percentage of patients with poor outcomes in this study. Still, the current study reports on one of the longest follow-ups in a large patient cohort. Second, the follow-up period was limited by the availability of the patient documentation. Therefore, no even longer follow-up could be obtained. Third, the quality of the co-morbidities was limited to the statements provided by the patients and patient records. Therefore, parameters such as BMI, ASA and smoking could not be assessed adequately. In order to compensate for possible missing data, the authors decided to facilitate the CCI and mFCI to assess a possible influence of the co-morbidities on the patient-reported outcome. Fourth, the qualitative 6-point Quality-of-Reduction scale showed only fair interrater reliability (κ = 0.32) and remains unvalidated, which limits confidence in analyses that use QoR as a predictor. Finally, the QoR was only subjective and based on radiographs as no postooperative CT images were available.

## Conclusion

At a mean follow-up of 18 years, patient-reported outcomes were related to the number of fractured malleoli. Moderate to poor results were uncommon after unimalleolar fractures (approximately 5%) but increased to approximately 30% after trimalleolar injuries; the loss to follow-up and retrospective design warrant cautious interpretation. Future studies should aim to include additional cofounders to identify further treatment targets.

## Supplemental Material

sj-pdf-1-fai-10.1177_10711007251361509 – Supplemental material for Long-term Functional Outcomes After Operatively Treated Unimalleolar, Bimalleolar, and Trimalleolar Ankle Fractures: A 15-22-Year Follow-up Study of 125 Patients Supplemental material, sj-pdf-1-fai-10.1177_10711007251361509 for Long-term Functional Outcomes After Operatively Treated Unimalleolar, Bimalleolar, and Trimalleolar Ankle Fractures: A 15-22-Year Follow-up Study of 125 Patients by Anne Scheuer, Fabian T. Spindler, Judith Schrempf, Wolfang Böcker, Hans Polzer and Sebastian F. Baumbach in Foot & Ankle International

sj-xlsx-2-fai-10.1177_10711007251361509 – Supplemental material for Long-term Functional Outcomes After Operatively Treated Unimalleolar, Bimalleolar, and Trimalleolar Ankle Fractures: A 15-22-Year Follow-up Study of 125 Patients Supplemental material, sj-xlsx-2-fai-10.1177_10711007251361509 for Long-term Functional Outcomes After Operatively Treated Unimalleolar, Bimalleolar, and Trimalleolar Ankle Fractures: A 15-22-Year Follow-up Study of 125 Patients by Anne Scheuer, Fabian T. Spindler, Judith Schrempf, Wolfang Böcker, Hans Polzer and Sebastian F. Baumbach in Foot & Ankle International
